# Mice and minipigs with compromised expression of the Alzheimer’s disease gene *SORL1* show cerebral metabolic disturbances on hyperpolarized [1-^13^C]pyruvate and sodium MRI

**DOI:** 10.1093/braincomms/fcae114

**Published:** 2024-03-31

**Authors:** Nikolaj Bøgh, Charlotte B Sørensen, Aage K O Alstrup, Esben S S Hansen, Olav M Andersen, Christoffer Laustsen

**Affiliations:** Department of Clinical Medicine, The MR Research Centre, Aarhus University, 8200 Aarhus, Denmark; A&E, Gødstrup Hospital, 7400 Herning, Denmark; Department of Clinical Medicine, Aarhus University, 8200 Aarhus, Denmark; Department of Clinical Medicine, Aarhus University, 8200 Aarhus, Denmark; Department of Nuclear Medicine and PET-Centre, Aarhus University Hospital, 8200 Aarhus, Denmark; Department of Clinical Medicine, The MR Research Centre, Aarhus University, 8200 Aarhus, Denmark; Department of Biomedicine, Aarhus University, 8200 Aarhus, Denmark; Department of Clinical Medicine, The MR Research Centre, Aarhus University, 8200 Aarhus, Denmark

**Keywords:** MRI, metabolism, Alzheimer’s disease, SORLA, *SORL1*

## Abstract

The sortilin-related receptor 1 (*SORL1*) gene, encoding the cellular endosomal sorting-related receptor with A-type repeats (SORLA), is now established as a causal gene for Alzheimer’s disease. As the latest addition to the list of causal genes, the pathophysiological effects and biomarker potential of *SORL1* variants remain relatively undiscovered. Metabolic dysfunction is, however, well described in patients with Alzheimer’s disease and is used as an imaging biomarker in clinical diagnosis settings. To understand the metabolic consequences of loss-of-function *SORL1* mutations, we applied two metabolic MRI technologies, sodium (^23^Na) MRI and MRI with hyperpolarized [1-^13^C]pyruvate, in minipigs and mice with compromised expression of *SORL1*. At the age analysed here, both animal models display no conventional imaging evidence of neurodegeneration but show biochemical signs of elevated amyloid production, thus representing the early preclinical disease. With hyperpolarized MRI, the exchange from [1-^13^C]pyruvate to [1-^13^C]lactate and ^13^C-bicarbonate was decreased by 32 and 23%, respectively, in the cerebrum of *SORL1*-haploinsufficient minipigs. A robust 11% decrease in the sodium content was observed with ^23^Na-MRI in the same minipigs. Comparably, the brain sodium concentration gradually decreased from control to *SORL1* haploinsufficient (−11%) to *SORL1* knockout mice (−23%), suggesting a gene dose dependence in the metabolic dysfunction. The present study highlights that metabolic MRI technologies are sensitive to the functional, metabolic consequences of Alzheimer’s disease and Alzheimer’s disease–linked genotypes. Further, the study suggests a potential avenue of research into the mechanisms of metabolic alterations by *SORL1* mutations and their potential role in neurodegeneration.

## Teaser

Bøgh *et al.* report that mice and pigs with compromised expression of *SORL1*, an Alzheimer’s disease causal gene encoding an endosomal sorting receptor, show decreased cerebral metabolism and sodium contents using hyperpolarized carbon and sodium MRI, providing insight into the metabolic consequences of Alzheimer’s disease–linked genotypes.

## Graphical Abstract

**Graphical Abstract:**
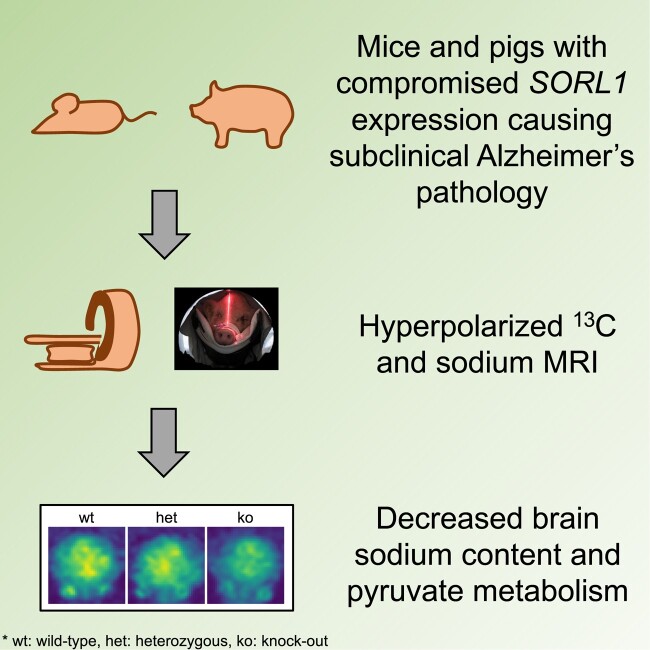

## Introduction

Alzheimer’s disease is the most common form of dementia.^1^ It is characterized by widespread, progressive loss of neurons, the basis of which is still being debated. The amyloid hypothesis dominates our understanding of Alzheimer’s disease, proposing that deposition of amyloid beta (Aβ) plaques causes the neurodegeneration. Several supplementing hypotheses point to other relevant pathophysiological mechanisms, including defects in the endosome recycling pathway.^2^ These alternative explanations have become increasingly important with intensifying debate over the amyloid hypothesis following recent results of amyloid-lowering clinical trials.^3^

The sortilin-related receptor 1 (*SORL1*) gene is now considered a causal gene for Alzheimer’s disease alongside with *APP*, *PSEN1* and *PSEN2.*^4^ It codes for the endosomal sorting receptor SORLA, and its deficiency causes dysfunction of the retromer complex, which is an important intracellular sorting and recycling system.^5,6^ The resulting endosomal traffic jam is associated with accumulation of Aβ,^7^ and endosomal recycling is thus important to consider in supplement to the classical understanding of Alzheimer’s disease pathophysiology.^2^ Recent research has suggested that loss-of-function *SORL1* mutations can be causative, meaning that they not just increase the risk of Alzheimer’s disease, but can cause Alzheimer’s disease pathology directly.^4,8^ However, close to 80% of the genetic variants identified in *SORL1* are missense variants, where risk effect can vary from benign to established pathogenic.^9^ As we are only starting to understand which of these variants are associated with high risk for Alzheimer’s disease, it is key to develop methods that can be used to monitor *SORL1* activity *in vivo*.

The endosomes have a multitude of vital roles within the cell. Therefore, in addition to being directly involved in Alzheimer’s disease pathology, endosomal dysfunction is expected to have widespread effects on the cellular apparatus. Metabolic dysfunction is emerging as a potential contributor to neurodegeneration in Alzheimer’s disease. This idea has spun from the extensive cortical hypometabolism observed with fluorodeoxyglucose PET (FDG-PET) in patients with Alzheimer’s disease.^10^ Though still a young field of research, a significant overlap has been observed between risk factors and assumed pathophysiological mechanisms of Type 2 diabetes mellitus and Alzheimer’s disease.^11^ The Alzheimer’s disease brain seems to be insulin resistant, even in the absence of Type 2 diabetes mellitus. This has led to the popular proposal of investigating Alzheimer’s disease as a Type 3 diabetes mellitus.^12^ Interestingly, SORLA has been shown to be a regulator of metabolism, as it recycles internalized insulin receptors to the plasma membrane, in turn increasing insulin sensitivity.^13^ Therefore, neuronal SORLA is a potential link between metabolic dysfunction and neurodegeneration in Alzheimer’s disease.

While metabolism is considered a potential contributor to neurodegeneration, the metabolic effects of Alzheimer’s disease mutations in the brain have been studied very little. Metabolic imaging provides a minimally invasive means of investigating brain metabolism *in vivo.* Among these, MRI with hyperpolarized [1-^13^C]pyruvate and MRI of ^23^Na represent sensitive readouts of disturbances to cellular metabolism.^14,15^ Hyperpolarized MRI relies on augmentation of the signal from [1-^13^C]pyruvate through hyperpolarization, whereby a metabolically active imaging probe is made.^16^ The pyruvate is then injected intravenously, and the pyruvate and its exchange with lactate and bicarbonate are imaged. Conversely, ^23^Na-MRI uses the natural abundance of sodium for imaging, reflecting the combined extra- and intracellular pools of sodium and the heavily energy-demanding processes sustaining these.^17^ In this study, we aimed to investigate brain metabolism in mice and minipigs genetically engineered to display reduced levels or complete absence of SORLA due to haploinsufficiency or full knockout of the *SORL1* gene. We hypothesized that decreased *SORL1* activity may display as dysfunctional metabolism observable with ^23^Na and hyperpolarized [1-^13^C]pyruvate MRI.

## Materials and methods

### Animals

This study included Göttingen minipigs (*n* = 12, 22–27 months of age) and mice (*n* = 27, 8 weeks of age) with reduced expression of *SORL1*. *SORL1* knockout (*ko*), heterozygous (*het*) and wild-type (*wt*) mice were bred from a line described in a previous study.^7^ The minipigs, all wild-type or *SORL1* heterozygous, where obtained from a recently described cohort based on somatic cell nuclear transfer cloning. The heterozygous genotype was shown to reflect the biochemical profile and *SORL1* expression of patients with Alzheimer’s disease with pathological *SORL1* variants.^8^ Mice and minipigs of both sexes were used (mice were 14/13 female/male, minipigs were 6/6 female/male), but the study was not powered to detect sex differences. The minipigs were housed as single animals in enriched pens with unrestricted access to water. They were fed a restricted diet (SDS Diet, UK). The mice were kept in small groups in enriched cages with unrestricted access to water and food. Both minipigs and mice were housed in 12/12 h of light/darkness at 20–22 °C and ∼55% humidity. They were allowed to acclimatize for at least 7 days before imaging. The experiments were approved by the Danish Animal Inspectorate.

### Magnetic resonance imaging of minipigs

The minipigs were fasted overnight (16 h) and premedicated with intramuscular s-ketamine (6.3 mg/kg) and midazolam (1.3 mg/kg). An ear vein catheter was placed for continuous administration of propofol, mechanical ventilation was initiated and the animals were moved to the MRI system (MR750, GE Healthcare). The minipigs underwent a full structural MRI protocol, the results of which are described elsewhere.^8^ Hereafter, the coil setup was changed to a custom-built 14-channel ^13^C-revieve coil with a commercial volume transmit (RAPID Biomedical). Anatomical images for reference were acquired using the integrated body coil of the scanner (T_1_-weighted 2D fast spin echo sequence, TR/TE = 807/6 ms, field of view = 400 mm^2^, matrix size = 260 × 280, slice thickness = 7.5 mm). Hyperpolarized [1-^13^C]pyruvate was produced using dissolution dynamic nuclear polarization^16^ in a commercial polarizer (SPINLab, GE Healthcare). The sample was dissoluted, buffered and diluted to a [1-^13^C]pyruvate concentration of 250 mM after polarizing for >2 h. The hyperpolarized pyruvate was rapidly administered through the ear vein (0.86 ml/kg at ∼5 ml/s) and chased with saline (20 ml at ∼5 ml/s). Imaging was performed as previously described.^18,19^ Briefly, spectral-spatial excitation was performed with a stack-of-spirals readout over four slices. The flip angles were 6°/37°/37° for pyruvate, lactate and bicarbonate, respectively. Time resolutions were 960 ms for pyruvate and 2880 ms for the metabolites. The spatial resolution was 1 × 1 × 1.5 cm^3^.

After hyperpolarized ^13^C-imaging, a pair of Helmholz loop ^23^Na-coils (PulseTeq Limited) was installed. Structural images for reference were obtained as above. Then, sodium images were acquired using a 3D sequence with a density-adapted radial readout (field of view = 350 mm^3^, matrix size = 35, flip angle = 20°, TR/TE = 5.3/0.3 ms, 16 averages). After imaging, the minipigs were returned to the housing facilities.

### Sodium magnetic resonance imaging of mice

The mice were anaesthetized with sevoflurane (6% for induction, ∼3–4% for maintenance) in medical air (1.5 l/min) through a nose cone. Imaging was performed using a 9.4 T preclinical system (Agilent Technologies) equipped with a 20 mm ^23^Na-loop coil. Sodium images were acquired using a 2D gradient echo sequence (field of view = 30 mm^2^, matrix size = 32, slice thickness = 20 mm, TR/TE = 15/1 ms, flip angle = 50°, 512 averages). After imaging, the mice were sacrificed by cervical dislocation under deep anaesthesia.

### Image processing and analysis

The raw data were gridded and fast Fourier transformed in *MATLAB* (MathWorks). The ratiometric approach was used for quantification of the ^13^C-data,^20^ while the raw signal normalized to the cerebrospinal fluid of the lateral ventricle or the aqueous humour of the eye was used for the ^23^Na-data.^21^ The ^13^C-data were zero-filled to 128 × 128 for display. Manual regions-of-interest were drawn to analyse the entire cerebrum, the frontal lobe, the temporal lobe and the cerebellum in the minipigs. In the mice, only the whole brain was analysed. The analyst was blind to animal group.

### Statistics

Plots and statistical analyses were made in R.^22^ Significance tests were performed as *t*-tests or ANOVA after graphical confirmation of normality. No formal sample size estimation was performed. No corrections were made for multiple testing due to the exploratory nature of the study. Data in the text are reported as means ± standard deviation.

## Results

### Metabolism of hyperpolarized [1-^13^C]pyruvate is decreased in *SORL1*-heterozygous minipigs

**Figure 1:**
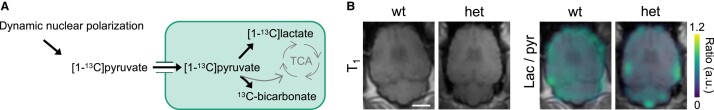
**Anatomical (T_1_) and metabolic MRI images of a wild-type (wt) and a *SORL1*-haploinsufficient minipigs (het).** Hyperpolarized [1-^13^C]pyruvate was polarized using dynamic nuclear polarization, enabling detection of [1-^13^C]pyruvate and its metabolites through their chemical shift (**A**). After injection of hyperpolarized [1-^13^C]pyruvate, images of lactate, bicarbonate and pyruvate are acquired, yielding maps of pyruvate-to-lactate and pyruvate-to-bicarbonate exchange (**B**). The white scale bar is ∼2 cm. a.u., arbitrary units; lac and pyr, lactate and pyruvate, respectively.

**Figure 2:**
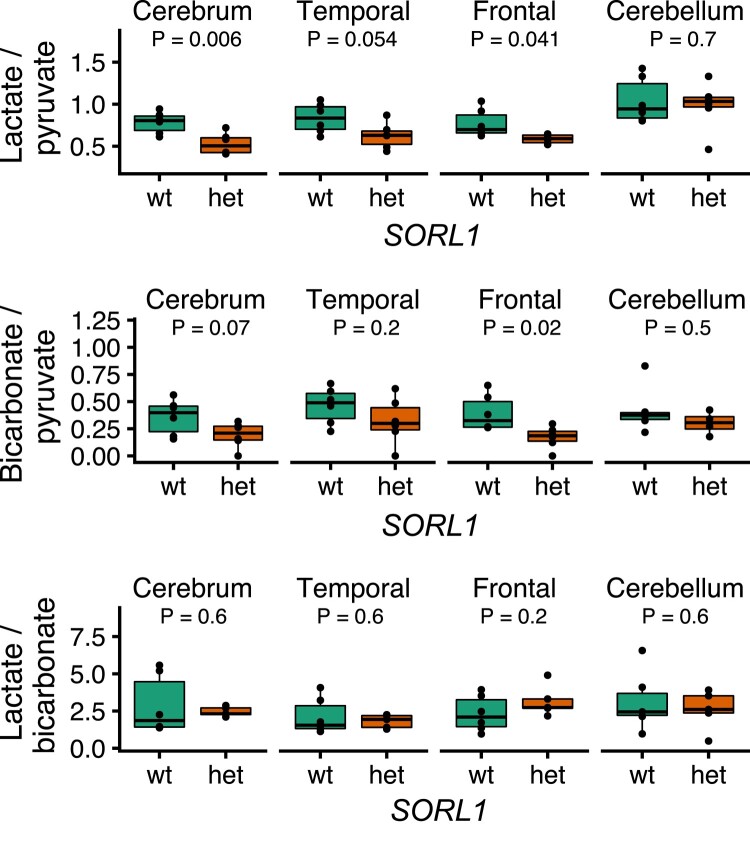
**Quantification of hyperpolarized MRI in minipigs with compromised *SORL1* expression.** The hyperpolarized [1-^13^C]pyruvate MRI data were quantified as ratios between pyruvate and the metabolites. Generally, pyruvate metabolism was decreased across the entire cerebrum as well as in frontal and temporal cortices. The ratios for the cerebellum were not different between wild-type (*wt*) and heterozygous (*het*) minipigs. Statistical significance was tested with *t*-tests, data points represent individual animals.

The signal-to-noise ratios of the ^13^C-data were 80 ± 43, 16.7 ± 10.9 and 5.8 ± 2.3 for pyruvate, lactate and bicarbonate, respectively. We used the model-free ratiometric approach as a simple measure of conversion of pyruvate to the downstream metabolites (Figs 1 and 2). The lactate-to-pyruvate ratio, reflecting pyruvate uptake and glycolysis, was decreased across the brain (0.53 ± 0.13 for *het* versus 0.78 ± 0.13 for *wt*, *P* = 0.006) and in the frontal cortex (0.59 ± 0.05 for *het* versus 0.77 ± 0.17 for *wt*, *P* = 0.04), while a trend was observed in the temporal cortex (0.63 ± 0.15 for *het* versus 0.83 ± 0.18 for *wt*, *P* = 0.05). No change was observed in the cerebellum, in line with our previous findings of undisturbed SORLA protein levels by the heterozygous gene expression in this region of the brain.^8^ The bicarbonate-to-pyruvate ratio, indicative of pyruvate uptake and oxidative metabolism, was only significantly altered in the frontal cortex (0.59 ± 0.1 for *het* versus 0.77 ± 0.17 for *wt*, *P* = 0.02). The lactate-to-bicarbonate ratio, reflecting the balance between pyruvate-to-lactate and pyruvate-to-bicarbonate metabolism, was similar between *wt* and *SORL1 het* minipigs.

### Sodium MRI signal is decreased in the cerebrum of *SORL1*-heterozygous minipigs

**Figure 3:**
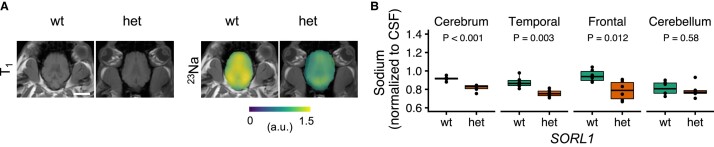
**Sodium MRI of wild-type (*wt*) or *SORL1* haploinsufficient minipigs (*het*).** The normalized sodium signal (**A**) was decreased in the brain of *SORL1*-compromised minipigs (*het*), while no difference between *het* and *wt* animals was observed in the cerebellum (**B**). Extracerebral signal was cropped for display. *T*-tests were used for assessment of statistical significance; data points represent individual animals. The white bar is ∼4 cm. a.u., arbitrary units; CSF, cerebrospinal fluid.

In addition to hyperpolarized ^13^C-MRI, we acquired ^23^Na-MRI (Fig. 3) which is sensitive to energy failure and the resulting ion transport disturbances.^23,24^ Here, we found a decrease in the total whole-brain sodium signal normalized to the CSF in the *SORL1*-compromised minipigs (0.82 ± 0.03 for *het* versus 0.92 ± 0.02 for *wt*, *P* = 0.0003), the frontal cortex (0.79 ± 0.1 for *het* versus 0.95 ± 0.07 for *wt*, *P* = 0.01) and the temporal cortex (0.76 ± 0.04 for *het* versus 0.88 ± 0.06 or *wt*, *P* = 0.003). Again, the cerebellum was not different between the two groups (0.79 ± 0.08 versus 0.81 ± 0.07, *P* = 0.6). Normalization of the signal from the eyes did not alter the results considerably (not shown).

### Sodium signal shows gene dose-dependent decrease in *Sorl1*-compromised mice

**Figure 4:**
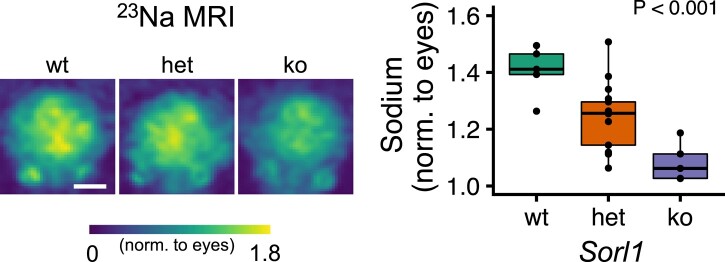
**Sodium MRI images of wild-type (*wt*) mice and mice heterozygous (*het*) or homozygous (*ko*) for *Sorl1*.** The normalized sodium signal across the brain was decreased in a gene dose-dependent manner. Statistical significance was assessed using ANOVA, data points represent individual animals, and the white scale bar is ∼5 mm.

Finally, we also examined the effects of *Sorl1* expression levels on ^23^Na-MRI in mice, which further allowed us to explore any gene dose-dependent differences between heterozygous and complete *Sorl1* knockout animals. We found that the total sodium signal normalized to the aqueous humour was decreased in the *het* and *ko* mice compared to *wt* (1.08 ± 0.07 for *ko* versus 1.25 ± 0.11 for *het* versus 1.41 ± 0.09 for *wt*, *P* = 0.0002). Importantly, this relationship was dependent on the gene dosage of *Sorl1* (Fig. 4), albeit with considerable variation in the *Sorl1 het* animals.

## Discussion

We studied the brain metabolic effects in genetically altered mice and minipigs with compromised expression of the Alzheimer’s disease gene *SORL1* using ^23^Na-MRI and hyperpolarized [1-^13^C]pyruvate MRI. Altered metabolism was observed in Alzheimer’s disease–relevant areas with both imaging technologies and in both species. Importantly, no changes were observed in the cerebellum of the minipigs, in accordance with this region expressing *SORL1* at normal levels.^8^ This serves as an internal control that the sodium concentration change was not due to a systemic alteration, and it suggests that the metabolic changes and the decrease in sodium follow the expression level of *SORL1*.

Hyperpolarized [1-^13^C]pyruvate MRI of the brain is currently being evaluated in several clinical trials.^14^ The focus is predominantly on applications in oncology, and just one case report has been published in neurodegenerative disease, suggesting feasibility of imaging the defective metabolism of the brain in amyotrophic lateral sclerosis.^25^ Moreover, to the best of our knowledge, no studies have evaluated hyperpolarized MRI in animal models of Alzheimer’s disease. Two reports demonstrated increased lactate-to-pyruvate ratios in animal models of multiple sclerosis, likely stemming from the large lactate production of proliferating immune cells.^26,27^ As multiple sclerosis, contrary to Alzheimer’s disease, primarily an inflammatory disease, their results and the data presented here suggest that Alzheimer’s disease–disposing *SORL1* mutations and neuroinflammation lead to opposite changes in the pyruvate-to-lactate exchange. It is important to notice that the *SORL1-*heterozygous minipigs did not display neurodegenerative changes on histology or structural imaging, but that the animals displayed amyloid build-up in the cerebrospinal fluid.^8^

**Figure 5:**
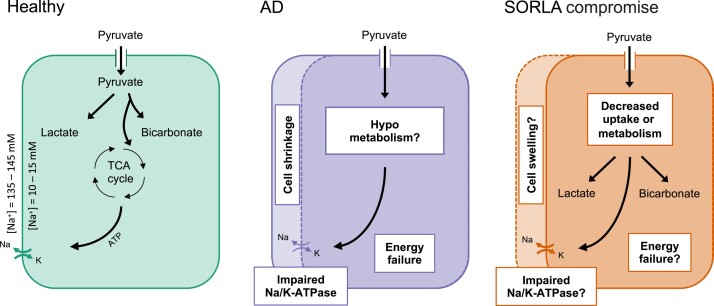
Schematic of the imaged metabolic changes in *SORL1*-compromised animals and potential underlying mechanisms in comparison with Alzheimer’s disease. The energy requirements of a healthy brain are fulfilled by glucose oxidation and glycolysis, and much of this energy is spend on maintaining the sodium gradient across the cell membrane. Hypometabolism is a typical feature of Alzheimer’s disease, hypothesized to cause failure in maintaining the sodium gradient and, in combination with cell death, leading to an increased ^23^Na-MRI signal.^23,24^ In the *SORL1*-compromised animals of this study, we observed a decrease in pyruvate-to-lactate and pyruvate-to-bicarbonate conversion coupled with a decrease in apparent ^23^Na. The former could be due to decreased pyruvate uptake or metabolism, while the latter could be caused by cellular volume changes regardless of energy status. TCA, tricarboxylic acid cycle.

The cellular mechanisms behind the metabolic changes that we observe may be manyfold (Fig. 5). Changes in *SORL1* expression lead to widespread changes in the transcriptome in young animals.^28,29^ The expression of the lactate dehydrogenases and of the monocarboxylate transporters, which transport pyruvate across the blood–brain barrier and lactate between neurons and astrocytes, is decreased in mouse and rat models of Alzheimer’s disease.^30,31^ This may be directly related to *SORL1* expression, as the retromer complex is known to recycle the monocarboxylate transporters to the cell membrane.^32^ Decreased levels of cellular pyruvate uptake with intact vascular delivery would explain the equivalent decrease of pyruvate-to-lactate and pyruvate-to-bicarbonate exchange that we observed as the vascular pyruvate signal is significant. This is further shown by similar lactate/bicarbonate ratios between the two groups. Mounting evidence suggests that, in some cases, dysfunction of the metabolic interplay between neurons and glial cells may underlie neurodegeneration.^30,33^ The ability of hyperpolarized MRI to resolve the downstream metabolites of pyruvate may allow probing of this.^34^ Lastly, the decreased conversion from pyruvate to its metabolites may also be due to effects further upstream, such as impaired capillary function or blood–brain barrier function leading to decreased cellular pyruvate uptake.^35^ Due to the complexity of the biology involved in hyperpolarized [1-^13^C]pyruvate imaging of the brain, the exact mechanisms behind the metabolic alterations we observe are elusive, but are of interest to the fields of Alzheimer’s disease and hyperpolarized MRI and should warrant further investigations.

In addition to changes in pyruvate metabolism, we observed altered levels of brain sodium signal in both mice and minipigs with decreased *SORL1* expression. Changes observed with ^23^Na-MRI are classically thought to reflect dysfunction of the Na/K-ATPase due to energy deprivation or shifts in the volume fractions between the extra- and intracellular spaces.^23,24^ To our surprise, we observed a decrease in the sodium signal in animals with compromised *SORL1* expression. This is contrary to the available imaging and biochemical data from confirmed patients with Alzheimer’s disease and other neurodegenerative diseases.^36-43^ Of note here, cerebral SORLA levels, sodium handling and metabolism in general are poorly understood at the earliest stages of Alzheimer’s disease that is most likely to correspond to the applied *in vivo* models. Even though rarely reported, decreases in ^23^Na-MRI signals within the brain are observed. For example, Gerhalter *et al.*^44^ found decreased brain sodium signal in mild traumatic brain injury patients, and similar observations have been made in the initial phase of animal models of stroke.^45,46^ It is speculated that the sodium decrease is a consequence of cell swelling, which could lead to a decrease in the sodium signal despite potential intracellular concentration increases caused by energy deprivation.^44,46^ Endosomes and axons swell in early Alzheimer’s disease,^5,47^ also reflected in the minipig model,^8^ suggesting that similar processes could drive the sodium decrease that we observed. Another cause of the increased intracellular volume fraction could be the accumulation of glial cells observed in Alzheimer’s disease.^48^ Alternatively, as sodium channels are readily trafficked and recycled by the retromer complex, the decreased sodium signal could be driven by endosomal failure due to the decrease of *SORL1* activity, leading to suboptimal cellular distribution of sodium channels.^32,49^ Based on our findings, we are, however, unable to anything but speculate on the exact mechanisms and their *SORL1* dependency. Future experiments in cells or with imaging chemical shift reagents may be useful in this regard.^24^

In the interpretation of our data, it is important to recognize that the animals used in this study do not reflect the pathologies of patients with Alzheimer’s disease at the time of diagnosis. They were young animals, and neither model display Aβ disposition in plaques at this age nor are they showing major signs of brain atrophy.^7,8,50^ They were likely in what has been termed the cellular, subclinical or biochemical phase of Alzheimer’s disease, where dysfunction in several cellular processes, including some metabolic, precedes structural changes.^51^ The findings presented here, and their discrepancy from other reports, could also be explained solely by the genotype of the models. This is supported by the gene dose dependency observed in mice and by the fact that we observed no changes in the cerebellum of the minipigs, which display normal levels of SORLA.^8^ Of interest, a recent study reported a strong positive correlation between *SORL1* expression and metabolism across cognitively normal people and patients with Alzheimer’s disease.^52^ Additionally, age could play a role, and it is possible that these models would display increased sodium levels comparable with the published human data if the animals were older.^37,38^ In fact, due to the somewhat combined and sometimes opposite effects of changes to intracellular sodium concentrations and volume fractions, it is also possible that decreased sodium levels are a general feature of Alzheimer’s disease at very early stages, before they then increase above normal as the energy deficit becomes more severe. Unfortunately, with our current animal cohorts, we were unable to study these potential longitudinal effects and compare them to other Alzheimer’s disease models, even though that would be of interest.

Being research technologies that have not yet translated to routine clinical use, there is still room for improvement and development of hyperpolarized [1-^13^C]pyruvate and sodium MRI. The relatively coarse resolution of especially hyperpolarized [1-^13^C]pyruvate MRI introduces some uncertainties when analysing smaller regions close to large vessels, such as the occipital cortex. Both metabolic MRI technologies offer a deeper and different perspective on metabolism than FDG-PET, the current clinical standard. As shown in previously published data, a subset of the minipigs in this study did not display hypometabolism on FDG-PET,^8^ even though we found decreases in sodium and the pyruvate-to-lactate exchange. This suggests that metabolic MRI could be sensitive to the earliest cellular and metabolic changes in Alzheimer’s disease. But, apart from this, few head-to-head comparisons have been made between FDG-PET and metabolic MRI, and we are not aware of any studies addressing this issue in neurodegenerative diseases. As such, the clinical utility of the extended information available with metabolic MRI of hyperpolarized [1-^13^C]pyruvate and ^23^Na is yet to be explored. As the functional consequences of much of the rare missense *SORL1* variants are unknown,^9^ we speculate whether metabolic MRI might assist the evaluation of variant pathogenicity for carriers of *SORL1* variants of unknown significance. Sodium and hyperpolarized [1-^13^C]pyruvate MRI could then further be useful as an early readout of the effect of treatments that target metabolism or *SORL1*-directed treatment, given that metabolism and biochemistry are thought to change well before structure.^51^

## Conclusion

Our data support that *SORL1* has a plethora of effects on cellular metabolism many of which, independently or combined, could lead to neurodegeneration. Sodium and hyperpolarized [1-^13^C]pyruvate MRI offer a window into the metabolic consequences of *SORL1*-associated Alzheimer’s disease and the temporal changes during the development of disease. From here, further work is needed to evaluate the clinical potential of metabolic MRI in Alzheimer’s disease and to elucidate the mechanisms responsible for the observed metabolic alterations, including their genotype specificity and temporal dependence.

## Supplementary Material

fcae114_Supplementary_Data

## Data Availability

The presented data are available upon reasonable request to the corresponding author.
