# Supplementary material for: Mice and minipigs with compromised expression of the Alzheimer’s disease gene SORL1 show cerebral metabolic disturbances on hyperpolarized [1-13C]pyruvate and sodium MRI
Source: Brain Commun. 2024 Mar 31;6(2):fcae114. doi: 10.1093/braincomms/fcae114 (PMC11034025; doi:10.1093/braincomms/fcae114)
Supplement: fcae114_Supplementary_Data [file fcae114_supplementary_data.zip › Original Submission.pdf]

OXFORD UNIVERSITY PRESS | Brain Communications

SORLA-deficient mice and pigs show cerebral metabolic disturbances on 23Na and hyperpolarized [1-13C]pyruvate MRI

Journal:	*Brain Communications*
Manuscript ID	BRAINCOM-2023-555
Manuscript Type:	Original Article
Date Submitted by the Author:	30-Nov-2023
Complete List of Authors:	Bøgh, Nikolaj; Aarhus University, Department of Clinical Medicine; A&E Gødstrup Hospital<br>Sørensen, Charlotte B; Aarhus University, Department of Clinical Medicine<br>Alstrup, Aage; Aarhus Universitetshospital, Nuclear Medicine & PET<br>Hansen, Esben SS; Aarhus University, Department of Clinical Medicine<br>Andersen, Olav M.; Aarhus University, Department of Biomedicine<br>Laustsen, Christoffer; Aarhus University, MR Research Centre
Keywords:	MRI, metabolism, sodium, hyperpolarization, Alzheimer's Disease, SORLA

SCHOLARONE™ Manuscripts

SORLA-deficient mice and pigs show cerebral metabolic disturbances on ^{23}Na and hyperpolarized [1-^{13}C]pyruvate MRI

Nikolaj Bøgh[1,2,*], Charlotte B Sørensen[3], Aage KO Alstrup[3,4], Esben SS Hansen[1], Olav M Andersen[5], Christoffer Laustsen[1]

[1]: The MR Research Centre, Department of Clinical Medicine, Aarhus University, Denmark.
[2]: A&E, Gødstrup Hospital, Denmark.
[3]: Department of Clinical Medicine, Aarhus University, Denmark.
[4]: Department of Nuclear Medicine & PET-Centre, Aarhus University Hospital, Denmark.
[5]: Department of Biomedicine, Aarhus University, Denmark
*: Please address correspondence to nikolaj.boegh@clin.au.dk

Running title: Metabolic MRI of SORLA-deficient animals
Word count (introduction to references): 3214
Original article: Research Article

For Review Only

Abstract

The *SORL1*-gene, encoding the cellular endosomal sorting receptor SORLA, is now established as a causal gene of Alzheimer's Disease (AD). As the latest addition to the list of causal genes, the pathophysiological effects and biomarker potential of *SORL1*-variants remain relatively undiscovered. Metabolic dysfunction is, however, well described in patients with AD and is used as an imaging biomarker in clinical diagnosis settings. To understand the metabolic consequences of loss-of-function *SORL1*-mutations, we applied two metabolic magnetic resonance imaging (MRI) technologies, ^{23}Na-MRI and MRI with hyperpolarized [1-^{13}C]pyruvate, in minipigs and mice with insufficiency of *SORL1*. At the age analyzed here, both animal models display no conventional imaging evidence of neurodegeneration but show biochemical signs of elevated amyloid production, thus representing the early preclinical disease. With hyperpolarized MRI, the exchange from [1-^{13}C]pyruvate to [1-^{13}C]lactate and ^{13}C-bicarbonate was decreased by 32 % and 23 %, respectively, in the cerebrum of *SORL1* haploinsufficient pigs. A robust 11 % decrease in the sodium content was observed with ^{23}Na-MRI in the same pigs. Comparably, the brain sodium concentration gradually decreased from control to haploinsufficient (-11 %) to knockout mice (-23 %), suggesting a gene dose-dependence in the metabolic dysfunction. The present study highlights that metabolic MRI technologies are sensitive to the functional, metabolic consequences of AD and AD-linked genotypes. Further, the study suggests a potential avenue of research into the mechanisms of metabolic alterations by *SORL1*-mutations and their potential role in neurodegeneration.

Keywords: MRI, metabolism, sodium, hyperpolarization, Alzheimer's Disease, SORLA, *SORL1*, pre-clinical model

For Review Only

Introduction

Alzheimer's Disease (AD) is the most common form of dementia[1]. It is characterized by wide-spread, progressive loss of neurons, the basis of which is still being debated. The amyloid hypothesis dominates our understanding of AD proposing that deposition of beta amyloid (Aβ) plaques causes the neurodegeneration. Several supplementing hypotheses point to other relevant pathophysiological mechanisms, including defects in the endosome recycling pathway[2]. These alternative explanations have become increasingly important with intensifying debate over the amyloid hypothesis following recent results of amyloid-lowering clinical trials[3].

The sortilin related receptor 1 (*SORL1*) gene is now considered a causal gene for AD alongside with *APP*, *PSEN1* and *PSEN2*[4]. It codes for the endosomal sorting receptor SORLA, and its deficiency causes dysfunction of the retromer complex, which is an important intracellular sorting and recycling system[5,6]. The resulting endosomal traffic jam is associated with accumulation of Aβ[7], and endosomal recycling is thus important to consider in supplement to the classical understanding of AD pathophysiology[2]. Recent research has suggested that loss-of-function *SORL1*-mutations can be causative, meaning that they not just increase the risk of AD, but can cause AD-pathology directly[4,8]. However, close to 80% of the genetic variants identified in *SORL1* are missense variants, where risk effect can vary from benign to established pathogenic[9]. As we are only starting to understand which of these variants are associated with high risk for AD, it is key to develop methods that can be used to monitor *SORL1* activity *in vivo*.

The endosomes have a multitude of vital roles within the cell. Therefore, in addition to being directly involved in AD-pathology, endosomal dysfunction is expected to have widespread effects on the cellular apparatus. Metabolic dysfunction is emerging as a potential contributor to neurodegeneration in AD. This idea has spun from the extensive cortical hypometabolism observed

For Review Only

with fluorodeoxyglucose positron emission tomography (FDG-PET) in AD-patients[10]. Though still a young field of research, a significant overlap has been observed between risk factors and assumed pathophysiological mechanisms of type 2 diabetes mellitus and AD[11]. The AD-brain seems to be insulin resistant, even in the absence of type 2 diabetes mellitus. This has led to the popular proposal of investigating AD as a type 3 diabetes mellitus[12]. Interestingly, SORLA has been shown to be a regulator of metabolism, as it recycles internalized insulin receptors to the plasma membrane, in turn increasing insulin sensitivity[13]. Therefore, neuronal SORLA is a potential link between metabolic dysfunction and neurodegeneration in AD.

While metabolism is considered a potential contributor to neurodegeneration, the metabolic effects of AD-mutations in the brain have been studied very little. Metabolic imaging provides a minimally invasive means of investigating brain metabolism *in vivo.* Among these, MRI with hyperpolarized [1-^{13}C]pyruvate and MRI of ^{23}Na represent sensitive readouts of disturbances to cellular metabolism[14,15]. Hyperpolarized MRI relies on augmentation of the signal from [1-^{13}C]pyruvate through the process of hyperpolarization, whereby a metabolically active imaging probe is made[16]. The pyruvate is then injected intravenously, and the pyruvate and its exchange to lactate and bicarbonate are imaged. Conversely, ^{23}Na-MRI uses the natural abundance of sodium for imaging, reflecting the combined extra- and intracellular pools of sodium and the heavily energy-demanding processes sustaining these[17]. In this study, we aimed to investigate brain metabolism in mice and minipigs genetically engineered to display SORLA deficiency. We hypothesized that decreased *SORL1* activity may display as dysfunctional metabolism observable with ^{23}Na and hyperpolarized [1-^{13}C]pyruvate MRI.

For Review Only

Materials and Methods

Animals

This study included SORLA-deficient Göttingen minipigs (n = 12, 22 – 27 months of age) and mice (n = 27, 8 weeks of age). Knockout (*ko*), heterozygous (*het)*, and wild-type (*wt)* mice were bred from a line described in a previous study[7]. The pigs, all wild-type or *SORL1* heterozygous, where obtained from a recently described cohort based on somatic cell nuclear transfer cloning[8]. Mice and minipigs of both sexes were used. The pigs were housed as single animals in enriched pens with unrestricted access to water. They were fed a restricted diet (SDS Diet, UK). The mice were kept in small groups in enriched cages with unrestricted access to water and food. Both pigs and mice were housed in 12/12 hours of light/darkness at 20-22 °C and ~55 % humidity. They were allowed to acclimatize for at least seven days before imaging. The experiments were approved by the Danish Animal Inspectorate.

Magnetic resonance imaging of minipigs

The pigs were fasted overnight (16 h) and premedicated with intramuscular s-ketamine (6.3 mg/kg) and midazolam (1.3 mg/kg). An ear vein catheter was placed for continuous administration of propofol, mechanical ventilation was initiated, and the animals were moved to the MRI system (MR750, GE Healthcare). The minipigs underwent a full structural MRI protocol, the results of which are described elsewhere[8]. Hereafter, the coil setup was changed to a custom-built 14-channel ^{13}C-revieve coil with a commercial volume transmit (RAPID Biomedical). Anatomical images for reference were acquired using the integrated body coil of the scanner (T_1 weighted 2D fast spin echo sequence, TR/TE = 807/6 ms, field of view = 400 mm^2, matrix size = 260 × 280, slice thickness = 7.5 mm). Hyperpolarized [1-^{13}C]pyruvate was produced using dissolution dynamic nuclear

For Review Only

polarization[16] in a commercial polarizer (SPINLab, GE Healthcare). The sample was dissoluted, buffered and diluted to a [1-^{13}C]pyruvate concentration of 250 mM after polarizing for more than 2 hours. The hyperpolarized pyruvate was rapidly administered through the ear vein (0.86 ml/kg at ~5 ml/s) and chased with saline (20 ml at ~5 ml/s). Imaging was performed as previously described[18,19]. Briefly, spectral-spatial excitation was performed with a stack-of-spirals readout over four slices. The flip angles were 6°/37°/37° for pyruvate, lactate, and bicarbonate, respectively. Time resolutions were 960 ms for pyruvate and 2880 ms for the metabolites. The spatial resolution was 1 × 1 × 1.5 cm^3.

After hyperpolarized ^{13}C-imaging, a pair of Helmholz loop ^{23}Na-coils (PulseTeq Limited) was installed. Structural images for reference were obtained as above. Then, sodium images were acquired using a 3D sequence with a density-adapted radial readout (field of view = 350 mm^3, matrix size = 35, flip angle = 20°, TR/TE = 5.3/0.3 ms, 16 averages). After imaging, the pigs were returned to the housing facilities.

Sodium magnetic resonance imaging of mice

The mice were anaesthetized with sevoflurane (6 % for induction, ~3-4 % for maintenance) in medical air (1.5 l/min) through a nose cone. Imaging was performed using a 9.4 T preclinical system (Agilent Technologies) equipped with a 20 mm ^{23}Na-loop coil. Sodium images were acquired using a 2D gradient echo sequence (field of view = 30 mm^2, matrix size = 32, slice thickness = 20 mm, TR/TE = 15/1 ms, flip angle = 50°, 512 averages). After imaging, the mice were sacrificed by cervical dislocation under deep anesthesia.

Image processing and analysis

For Review Only

The raw data were gridded and fast Fourier transformed in *MATLAB* (MathWorks). The ratiometric approach was used for quantification of the ^{13}C-data[20], while the raw signal normalized to the cerebrospinal fluid of the lateral ventricle or the aqueous humor of the eye was used for the ^{23}Na-data[21]. The ^{13}C-data were zero-filled to 128 × 128 for display. Manual regions-of-interest were drawn to analyze the entire cerebrum, the frontal lobe, the temporal lobe, and the cerebellum in the pigs. In the mice, only the whole brain was analyzed. The analyst was blind to animal group.

Statistics

Plots and statistical analyses were made in R[22]. Significance tests were performed as T-tests or analysis of variance (ANOVA) after graphical confirmation of normality. No formal sample size estimation was performed. No corrections were made for multiple testing due to the exploratory nature of the study. Data in the text are reported as means ± standard deviation.

For Review Only

Results

Metabolism of hyperpolarized [1-^{13}C]pyruvate is decreased in SORL1 heterozygous minipigs

The signal-to-noise ratios of the ^{13}C-data were 80 ± 43, 16.7 ± 10.9, and 5.8 ± 2.3 for pyruvate, lactate, and bicarbonate, respectively. We used the model-free ratiometric approach as a simple measure of conversion of pyruvate to the downstream metabolites (Figures 1+2). The lactate-to-pyruvate ratio, reflecting pyruvate uptake and glycolysis, was decreased across the brain (0.53 ± 0.13 for *het* vs 0.78 ± 0.13 for *wt*, P = .006) and in the frontal cortex (0.59 ± 0.05 for *het* vs 0.77 ± 0.17 for *wt*, P = .04), while a trend was observed in the temporal cortex (0.63 ± 0.15 for *het* vs 0.83 ± 0.18 for *wt*, P = .05). No change was observed in the cerebellum, in line with our previous findings of undisturbed SORLA protein levels by the heterozygous gene expression in this region of the

brain[8]. The bicarbonate-to-pyruvate ratio, indicative of pyruvate uptake and oxidative metabolism, was only significantly altered in the frontal cortex (0.59 ± 0.1 for *het* vs 0.77 ± 0.17 for *wt*, P = .02). The lactate-to-bicarbonate ratio, reflecting the balance between glycolytic and oxidative metabolism, was similar between *wt* and *het* pigs.

Sodium MRI signal is decreased in the cerebrum of SORL1-heterozygous minipigs

In addition to hyperpolarized ^{13}C-MRI, we acquired ^{23}Na-MRI (Figure 3) which is sensitive to energy failure and the resulting ion transport disturbances[23,24]. Here, we found a decrease in the total whole-brain sodium signal normalized to the CSF in the *SORL1*-deficient animals (0.82 ± 0.03 for *het* vs 0.92 ± 0.02 for *wt*, P = .0003), the frontal cortex (0.79 ± 0.1 for *het* vs 0.95 ± 0.07 for *wt*, P = .01), and the temporal cortex (0.76 ± 0.04 for *het* vs 0.88 ± 0.06 or *wt*, P = .003). Again, the cerebellum was not different between the two groups (0.79 ± 0.08 vs 0.81 ± 0.07, P = .6). Normalization to the signal form the eyes did not alter the results considerably (not shown).

Sodium signal shows gene dose-dependent decrease in SORL1-deficient mice

Finally, we also examined the effects of *SORL1* expression levels on ^{23}Na-MRI in mice, which further allowed us to explore any gene dose-dependent differences between heterozygous and complete knock-out animals. We found that the total sodium signal normalized to the aqueous humor was decreased in the *het* and *ko* mice compared to *wt* (1.08 ± 0.07 for *ko* vs 1.25 ± 0.11 for *het* vs 1.41 ± 0.09 for *wt*, P = 0.0002). Importantly, this relationship was dependent on the dosage of *SORL1* (Figure 4), albeit with considerable variation in the *het* animals.

Discussion

For Review Only

We studied the brain metabolic effects of SORLA-deficiency in genetically altered mice and minipigs using ^{23}Na-MRI and hyperpolarized [1-^{13}C]pyruvate MRI. Altered metabolism was observed in AD-relevant areas with both imaging technologies and in both tested species. Importantly, no changes were observed in the cerebellum of the minipigs, in accordance with this region expressing *SORL1* at normal levels[8]. This serves as an internal control that the sodium concentration change was not due to a systemic alteration, and it suggest that the metabolic changes and the decrease in sodium follow the expression level of *SORL1*.

Hyperpolarized [1-^{13}C]pyruvate MRI of the brain is currently being evaluated in several clinical trials[14]. The focus is predominantly on applications in oncology, and just one case report has been published in neurodegenerative disease, suggesting feasibility of imaging the defective metabolism of the brain in amyotrophic lateral sclerosis[25]. Moreover, to the best of our knowledge, no studies have evaluated hyperpolarized MRI in animal models of AD. Two reports demonstrated increased lactate-to-pyruvate ratios in animal models of multiple sclerosis, likely stemming from the large lactate-production of proliferating immune cells[26,27]. As multiple sclerosis, contrary to AD, primarily is an inflammatory disease, their results and the data presented here suggest that AD-disposing *SORL1*-mutations and neuroinflammation lead to opposite changes in the pyruvate-to-lactate exchange. It is important to notice that the *SORL1*-deficient pigs did not display neurodegenerative changes on histology or structural imaging, but that the animals displayed amyloid build-up in the cerebrospinal fluid[8].

The cellular mechanisms behind the metabolic changes that we observe may be manyfold (Figure 5). Changes in *SORL1*-expression lead to widespread changes in the transcriptome in young animals[28,29]. The expression of the lactate dehydrogenases and of the monocarboxylate transporters, which transport pyruvate across the blood-brain barrier and lactate between neurons

For Review Only

and astrocytes, is decreased in mouse and rat models of AD[30,31]. This may be directly related to *SORL1* expression, as the retromer complex is shown to recycle the monocarboxylate transporters to the cell membrane[32]. Decreased levels of pyruvate uptake would explain the equivalent decrease of pyruvate-to-lactate and pyruvate-to-bicarbonate exchange that we observed. This is further shown by similar lactate/bicarbonate ratios between the two groups. Mounting evidence suggests that, in some cases, dysfunction of the metabolic interplay between neurons and glial cells may underlie neurodegeneration[30,33]. The ability of hyperpolarized MRI to resolve the downstream metabolites of pyruvate may allow probing of this[34]. Lastly, the decreased conversion from pyruvate to lactate may also be due to effects further upstream, such as impaired capillary function or blood-brain barrier function leading to decreased pyruvate delivery[35]. Due to the complexity of the biology involved in hyperpolarized [1-^{13}C]pyruvate imaging of the brain, the exact mechanisms behind the metabolic alterations we observe are elusive, but are of interest to the fields of AD and hyperpolarized MRI and should warrant further investigations.

In addition to changes in pyruvate metabolism, we observed altered levels of brain sodium signal in both mice and minipigs with decreased *SORL1* expression. Changes observed with ^{23}Na-MRI are classically thought to reflect dysfunction of the Na/K-ATPase due to energy deprivation or shifts in the volume fractions between the extra- and intracellular spaces[23,24]. To our surprise, we observed a decrease in the sodium signal in *SORL1*-deficient animals. This is contrary to the available imaging and biochemical data from confirmed AD patients and other neurodegenerative diseases[36–43]. Of note here, cerebral SORLA levels, sodium handling, and metabolism in general is poorly understood at the earliest stages of AD that is most likely to correspond to the here applied *in vivo* models. Even though rarely reported, decreases in ^{23}Na-MRI signals within the brain are observed. For example, Teresa et al found decreased brain sodium signal in mild traumatic brain injury

For Review Only

patients[44], and similar observations have been made in the initial phase of animal models of stroke[45,46]. It is speculated that the sodium decrease is a consequence of cell-swelling, which could lead to a decrease in the sodium signal despite potential intracellular concentration increases caused by energy deprivation[44,46]. Endosomes and axons swell in early AD[5,47], also reflected in the minipig model[8], suggesting that similar processes could drive the sodium decrease that we observed. Another cause of the increased intracellular volume fraction could be the accumulation of glial cells observed in AD[48]. Alternatively, as sodium channels are readily trafficked and recycled by the retromer-complex, the decreased sodium signal could be driven by endosomal failure due to the decrease of *SORL1* activity, leading to suboptimal cellular distribution of sodium channels[32,49]. Based on our findings we are, however, unable to anything but speculate on the exact mechanisms and their *SORL1*-dependency. Future experiments in cells or with imaging chemical shift reagents may be useful in this regard[24].

In the interpretation of our data, it is very important to recognize that the animals used in this study do not reflect the pathologies of diagnosed AD patients. They were young animals, and neither model is expected to display amyloid beta disposition in plaques at this age nor are they showing major signs of brain atrophy[7,8,50]. They were likely in what has been termed the cellular, preclinical, or biochemical phase of AD, where dysfunction in several cellular processes, including some metabolic, precedes structural changes[51]. The findings presented here, and their discrepancy from other reports, could also be explained solely by the genotype of the models. This is supported by the gene dose-dependency observed in mice, and by the fact that we observed no changes in the cerebellum of the pigs, which display normal levels of SORLA[8]. Of interest, a recent study reported a strong positive correlation between *SORL1*-expression and metabolism across cognitively normal people and AD patients[52]. Additionally, age could play a role, and it is possible that these models

For Review Only

would display increased sodium levels comparable to the published human data if the animals were older[37,38]. In fact, due to the somewhat combined and sometimes opposite effects of changes to intracellular sodium concentrations and volume fractions, it is also possible that decreased sodium levels are a general feature of AD at very early stages, before they then increase above normal as the energy deficit becomes more severe. Unfortunately, with our current animal cohorts, we were unable to study these potential longitudinal effects and compare them to other AD-models, even though that would be of interest.

Being research technologies that have not yet translated to routine clinical use, there is still room for improvement and development of hyperpolarized [1-^{13}C]pyruvate and sodium MRI. The relatively coarse resolution of especially hyperpolarized [1-^{13}C]pyruvate MRI introduces some uncertainties when analyzing smaller regions close to large vessels, such as the occipital cortex. Both metabolic MRI technologies offer a deeper and different perspective on metabolism than FDG-PET, the current clinical standard. As shown in previously published data, a subset of the minipigs in this study did not display hypometabolism on FDG-PET[8], even though we found decreases in sodium and the pyruvate-to-lactate exchange. This suggests that metabolic MRI could be sensitive to the earliest cellular and metabolic changes in AD. But, apart from this, few head-to-head comparisons have been made between FDG-PET and metabolic MRI, and we are not aware of any studies addressing this issue in neurodegenerative diseases. As such, the clinical utility of the extended information available with metabolic MRI of hyperpolarized [1-^{13}C]pyruvate and ^{23}Na is yet to be explored. As the functional consequences of much of the rare missense *SORL1* variants are unknown[9], we speculate whether metabolic MRI might assist the evaluation of variant pathogenicity for carriers of *SORL1* variants of unknown significance. Sodium and hyperpolarized [1-^{13}C]pyruvate MRI could then further be useful as an early readout of the effect of treatments that target metabolism or

For Review Only

SORL1-directed treatment, given that metabolism and biochemistry are thought to change well before structure[51].

In conclusion, our data support that *SORL1* have a plethora of effects on cellular metabolism many of which, independently or combined, could lead to neurodegeneration. Sodium and hyperpolarized [1-^{13}C]pyruvate MRI offer a window into the metabolic consequences of *SORL1*-associated AD and the temporal changes during the development of disease. From here, further work is needed to evaluate the clinical potential of metabolic MRI in AD, and to elucidate the mechanisms responsible for the observed metabolic alterations, including their genotype specificity and temporal dependence.

For Review Only

Data availability

The presented data are available upon reasonable request to the corresponding author.

Acknowledgements

The authors acknowledge Anne Mette V. Toft, Mette Bak, and Martin A. Fredsted for their expert assistance with animal handling. Further, they acknowledge Thomas Willnow from Aarhus University and Lars F. Mikkensen from Ellegaard Göttingen Minipigs A/S for supplying the animals.

Funding

This study was supported by grants from the Lundbeck Foundation (R100-A9209 and R272-2017-4023), The Danish Heart Association (16-R107-A6813-22997), the Ellegaard Göttingen Minipigs Research Foundation, and Ellegaard Göttingen Minipigs A/S.

Conflicts of Interest

O.M.A. has commercial interests in Retromer Therapeutics, but the company was not involved in any aspects of the study. Ellegaard Göttingen Minipigs A/S owns the commercial rights to the *SORL1* minipigs. N.B, O.M.A, C.B.S, and C.L. have filed a patent describing the use of sodium MRI for measuring in vivo *SORL1* activity.

References

1. Knopman DS, Amieva H, Petersen RC, et al. Alzheimer disease. *Nat Rev Dis Primers*. 2021;7(1):1-21. doi:10/gj2ns7
2. Small SA, Petsko GA. Endosomal recycling reconciles the Alzheimer's disease paradox. *Sci Transl Med*. 2020;12(572):eabb1717. doi:10.1126/scitranslmed.abb1717
3. Karran E, De Strooper B. The amyloid hypothesis in Alzheimer disease: new insights from new therapeutics. *Nat Rev Drug Discov*. 2022;21(4):306-318. doi:10.1038/s41573-022-00391-w
4. Raghavan NS, Brickman AM, Andrews H, et al. Whole-exome sequencing in 20,197 persons for rare variants in Alzheimer's disease. *Ann Clin Transl Neurol*. 2018;5(7):832-842. doi:10.1002/acn3.582
5. Knupp A, Mishra S, Martinez R, et al. Depletion of the AD Risk Gene SORL1 Selectively Impairs Neuronal Endosomal Traffic Independent of Amyloidogenic APP Processing. *Cell Rep*. 2020;31(9):107719. doi:10.1016/j.celrep.2020.107719
6. Mishra S, Knupp A, Szabo MP, et al. The Alzheimer's gene SORL1 is a regulator of endosomal traffic and recycling in human neurons. *Cell Mol Life Sci*. 2022;79(3):162. doi:10.1007/s00018-022-04182-9
7. Andersen OM, Reiche J, Schmidt V, et al. Neuronal sorting protein-related receptor sorLA/LR11 regulates processing of the amyloid precursor protein. *Proc Natl Acad Sci U S A*. 2005;102(38):13461-13466. doi:10.1073/pnas.0503689102

For Review Only

8. Andersen OM, Bøgh N, Landau AM, et al. A genetically modified minipig model for Alzheimer's disease with SORL1 haploinsufficiency. *CR Med*. 2022;3(9):100740. doi:10.1016/j.xcrm.2022.100740

9. Campion D, Charbonnier C, Nicolas G. SORL1 genetic variants and Alzheimer disease risk: a literature review and meta-analysis of sequencing data. *Acta Neuropathol*. 2019;138(2):173-186. doi:10.1007/s00401-019-01991-4

10. Brown RKJ, Bohnen NI, Wong KK, Minoshima S, Frey KA. Brain PET in Suspected Dementia: Patterns of Altered FDG Metabolism. *RadioGraphics*. 2014;34(3):684-701. doi:10.1148/rg.343135065

11. Arnold SE, Arvanitakis Z, Macauley-Rambach SL, et al. Brain insulin resistance in type 2 diabetes and Alzheimer disease: concepts and conundrums. *Nat Rev Neurol*. 2018;14(3):168-181. doi:10.1038/nrneurol.2017.185

12. Steen E, Terry BM, Rivera EJ, et al. Impaired insulin and insulin-like growth factor expression and signaling mechanisms in Alzheimer's disease--is this type 3 diabetes? *J Alzheimers Dis*. 2005;7(1):63-80. doi:10/gf2872

13. Schmidt V, Schulz N, Yan X, et al. SORLA facilitates insulin receptor signaling in adipocytes and exacerbates obesity. *J Clin Invest*. 2016;126(7):2706-2720. doi:10.1172/JCI84708

14. Vaeggemose M, F. Schulte R, Laustsen C. Comprehensive Literature Review of Hyperpolarized Carbon-13 MRI: The Road to Clinical Application. *Metabolites*. 2021;11(4):219. doi:10/gjnt6j

15. Grist JT, McLean MA, Riemer F, et al. Quantifying normal human brain metabolism using hyperpolarized [1-^{13}C]pyruvate and magnetic resonance imaging. *Neuroimage*. 2019;189:171-179. doi:10/ggsdsh

16. Ardenkjaer-Larsen JH, Fridlund B, Gram A, et al. Increase in signal-to-noise ratio of > 10,000 times in liquid-state NMR. *Proc Natl Acad Sci U S A*. 2003;100(18):10158-10163. doi:10/cmbvwf

For Review Only

17. Thulborn KR. Quantitative sodium MR imaging: A review of its evolving role in medicine. *NeuroImage*. 2018;168:250-268. doi:10.1016/j.neuroimage.2016.11.056

18. Bøgh N, Olin RB, Hansen ES, et al. Metabolic MRI with hyperpolarized [1- ^{13}C]pyruvate separates benign oligemia from infarcting penumbra in porcine stroke. *J Cereb Blood Flow Metab*. 2021;41(11):2916-2927. doi:10/gj4kfw

19. Olin RB, Sanchez-Heredia JD, Schulte RF, et al. Three-dimensional accelerated acquisition for hyperpolarized ^{13}C MR with blipped stack-of-spirals and conjugate-gradient SENSE. *Magn Reson Med*. Published online January 20, 2020. doi:10/ggsdt4

20. Hill DK, Orton MR, Mariotti E, et al. Model Free Approach to Kinetic Analysis of Real-Time Hyperpolarized ^{13}C Magnetic Resonance Spectroscopy Data. *PLOS ONE*. 2013;8(9):e71996. doi:10/f5gc57

21. Adlung A, Licht C, Reichert S, et al. Quantification of tissue sodium concentration in the ischemic stroke: A comparison between external and internal references for 23Na MRI. *Journal of Neuroscience Methods*. 2022;382:109721. doi:10.1016/j.jneumeth.2022.109721

22. R Core Team. R: A language and environment for statistical computing. R Foundation for Statistical Computing. Published online 2021. https://www.R-project.org/

23. Gerkau NJ, Rakers C, Petzold GC, Rose CR. Differential effects of energy deprivation on intracellular sodium homeostasis in neurons and astrocytes. *Journal of Neuroscience Research*. 2017;95(11):2275-2285. doi:10.1002/jnr.23995

24. Burstein D, Springer Jr CS. Sodium MRI revisited. *Magnetic Resonance in Medicine*. 2019;82(2):521-524. doi:10.1002/mrm.27738

25. Bøgh N, Laustsen C, Hansen ESS, Tankisi H, Bertelsen LB, Blicher JU. Imaging Neurodegenerative Metabolism in Amyotrophic Lateral Sclerosis with Hyperpolarized [1-^{13}C]pyruvate MRI. *Tomography*. 2022;8(3):1570-1577. doi:10.3390/tomography8030129

For Review Only

26. Guglielmetti C, Najac C, Didonna A, Van der Linden A, Ronen SM, Chaumeil MM. Hyperpolarized ^{13}C MR metabolic imaging can detect neuroinflammation in vivo in a multiple sclerosis murine model. *Proc Natl Acad Sci U S A*. 2017;114(33):E6982-E6991. doi:10/gbtb9p

27. Le Page LM, Guglielmetti C, Najac CF, Tiret B, Chaumeil MM. Hyperpolarized ^{13}C magnetic resonance spectroscopy detects toxin-induced neuroinflammation in mice. *NMR in Biomedicine*. 2019;32(11):e4164. doi:10/ggsgsz

28. Barthelson K, Pederson SM, Newman M, Lardelli M. Brain Transcriptome Analysis of a Protein-Truncating Mutation in Sortilin-Related Receptor 1 Associated With Early-Onset Familial Alzheimer's Disease Indicates Early Effects on Mitochondrial and Ribosome Function. *J Alzheimers Dis*. 2021;79(3):1105-1119. doi:10.3233/JAD-201383

29. Barthelson K, Pederson SM, Newman M, Lardelli M. Brain transcriptome analysis reveals subtle effects on mitochondrial function and iron homeostasis of mutations in the SORL1 gene implicated in early onset familial Alzheimer's disease. *Mol Brain*. 2020;13(1):142. doi:10.1186/s13041-020-00681-7

30. Zhang M, Cheng X, Dang R, Zhang W, Zhang J, Yao Z. Lactate Deficit in an Alzheimer Disease Mouse Model: The Relationship With Neuronal Damage. *J Neuropathol Exp Neurol*. 2018;77(12):1163-1176. doi:10.1093/jnen/nly102

31. Lu W, Huang J, Sun S, et al. Changes in lactate content and monocarboxylate transporter 2 expression in $A\beta_{25-35}$-treated rat model of Alzheimer's disease. *Neurol Sci*. 2015;36(6):871-876. doi:10.1007/s10072-015-2087-3

32. Steinberg F, Gallon M, Winfield M, et al. A global analysis of SNX27–retromer assembly and cargo specificity reveals a function in glucose and metal ion transport. *Nat Cell Biol*. 2013;15(5):461-471. doi:10.1038/ncb2721

33. Lee Y, Morrison BM, Li Y, et al. Oligodendroglia metabolically support axons and contribute to neurodegeneration. *Nature*. 2012;487(7408):443-448. doi:10/f344v6

For Review Only

34. Bøgh N, Grist JT, Rasmussen CW, et al. Lactate saturation limits bicarbonate detection in hyperpolarized ^{13}C-pyruvate MRI of the brain. *Magnetic Resonance in Medicine*. 2022;88(3):1170-1179. doi:10.1002/mrm.29290

35. Østergaard L, Aamand R, Gutiérrez-Jiménez E, et al. The capillary dysfunction hypothesis of Alzheimer's disease. *Neurobiology of Aging*. 2013;34(4):1018-1031. doi:10.1016/j.neurobiolaging.2012.09.011

36. Vitvitsky VM, Garg SK, Keep RF, Albin RL, Banerjee R. Na+ and K+ ion imbalances in Alzheimer's disease. *Biochimica et Biophysica Acta (BBA) - Molecular Basis of Disease*. 2012;1822(11):1671-1681. doi:10.1016/j.bbadis.2012.07.004

37. Mellon EA, Pilkinton DT, Clark CM, et al. Sodium MR Imaging Detection of Mild Alzheimer Disease: Preliminary Study. *AJNR Am J Neuroradiol*. 2009;30(5):978-984. doi:10.3174/ajnr.A1495

38. Haeger A, Bottlaender M, Lagarde J, et al. What can 7T sodium MRI tell us about cellular energy depletion and neurotransmission in Alzheimer's disease? *Alzheimers Dement*. 2021;17(11):1843-1854. doi:10.1002/alz.12501

39. Petracca M, Vancea RO, Fleysher L, Jonkman LE, Oesingmann N, Inglese M. Brain intra- and extracellular sodium concentration in multiple sclerosis: a 7 T MRI study. *Brain*. 2016;139(Pt 3):795-806. doi:10.1093/brain/awv386

40. Collorone S, Prados F, Kanber B, et al. Brain microstructural and metabolic alterations detected in vivo at onset of the first demyelinating event. *Brain*. 2021;144(5):1409-1421. doi:10.1093/brain/awab043

41. Krahe J, Dogan I, Didszun C, et al. Increased brain tissue sodium concentration in Friedreich ataxia: A multimodal MR imaging study. *NeuroImage: Clinical*. 2022;34:103025. doi:10.1016/j.nicl.2022.103025

For Review Only

42. Hattori N, Kitagawa K, Higashida T, et al. Cl−-ATPase and Na+/K+-ATPase activities in Alzheimer's disease brains. *Neuroscience Letters*. 1998;254(3):141-144. doi:10.1016/S0304-3940(98)00654-5

43. Mohamed SA, Herrmann K, Adlung A, et al. Evaluation of Sodium (23Na) MR-imaging as a Biomarker and Predictor for Neurodegenerative Changes in Patients With Alzheimer's Disease. *In Vivo*. 2021;35(1):429-435. doi:10.21873/invivo.12275

44. Gerhalter T, Chen AM, Dehkharghani S, et al. Global decrease in brain sodium concentration after mild traumatic brain injury. *Brain Communications*. 2021;3(2):fcab051. doi:10.1093/braincomms/fcab051

45. Bartha R, Lee TY, Hogan MJ, et al. Sodium T2*-weighted MR imaging of acute focal cerebral ischemia in rabbits. *Magn Reson Imaging*. 2004;22(7):983-991. doi:10.1016/j.mri.2004.02.004

46. Wetterling F, Gallagher L, Mullin J, et al. Sodium-23 magnetic resonance imaging has potential for improving penumbra detection but not for estimating stroke onset time. *J Cereb Blood Flow Metab*. 2015;35(1):103-110. doi:10/f6vgbq

47. Fiala JC. Mechanisms of amyloid plaque pathogenesis. *Acta Neuropathol*. 2007;114(6):551-571. doi:10.1007/s00401-007-0284-8

48. Pekny M, Pekna M. Reactive gliosis in the pathogenesis of CNS diseases. *Biochimica et Biophysica Acta (BBA) - Molecular Basis of Disease*. 2016;1862(3):483-491. doi:10.1016/j.bbadis.2015.11.014

49. Cusdin FS, Clare JJ, Jackson AP. Trafficking and cellular distribution of voltage-gated sodium channels. *Traffic*. 2008;9(1):17-26. doi:10.1111/j.1600-0854.2007.00673.x

50. Dodson SE, Andersen OM, Karmali V, et al. Loss of LR11/SORLA Enhances Early Pathology in a Mouse Model of Amyloidosis: Evidence for a Proximal Role in Alzheimer's Disease. *J Neurosci*. 2008;28(48):12877-12886. doi:10.1523/JNEUROSCI.4582-08.2008

For Review Only

51. De Strooper B, Karran E. The Cellular Phase of Alzheimer's Disease. *Cell*. 2016;164(4):603-615. doi:10.1016/j.cell.2015.12.056

52. Ye F, Funk Q, Rockers E, Shulman JM, Masdeu JC, Pascual B. In Alzheimer-prone brain regions, metabolism and risk-gene expression are strongly correlated. *Brain Commun*. 2022;4(5):fcac216. doi:10.1093/braincomms/fcac216

For Review Only

Figures

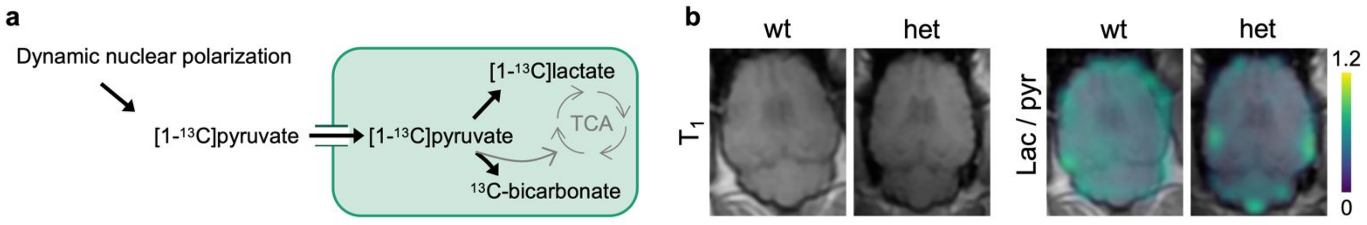

Figure 1: Anatomical (T_1) and metabolic MRI images of a wild type (wt) and a SORL1-haploinsufficient pig (het). Hyperpolarized [1-^{13}C]pyruvate was polarized using dynamic nuclear polarization, enabling detection of [1-^{13}C]pyruvate and its metabolites through their chemical shift (**a**). After injection of hyperpolarized [1-^{13}C]pyruvate, images of lactate, bicarbonate, and pyruvate are acquired, yielding maps of pyruvate-to-lactate and pyruvate-to-bicarbonate exchange (**b**).

For Review Only

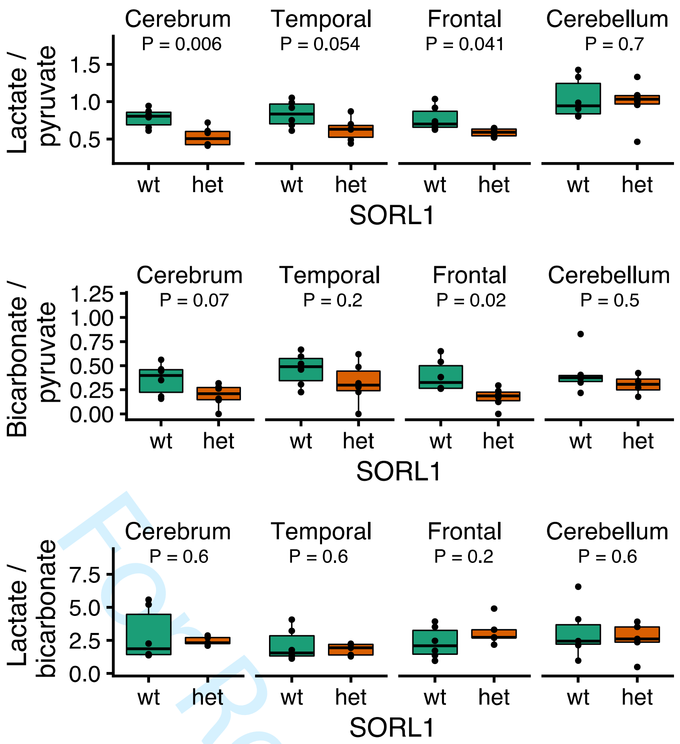

Figure 2: The hyperpolarized [1-^{13}C]pyruvate MRI data were quantified as ratios between pyruvate and the metabolites. Generally, pyruvate metabolism was decreased across the entire cerebrum as well as in frontal and temporal cortices. The ratios for the cerebellum were not different between wildtype (*wt*) and heterozygous (*het*) pigs. Statistical significance was tested with t-tests.

For Review Only

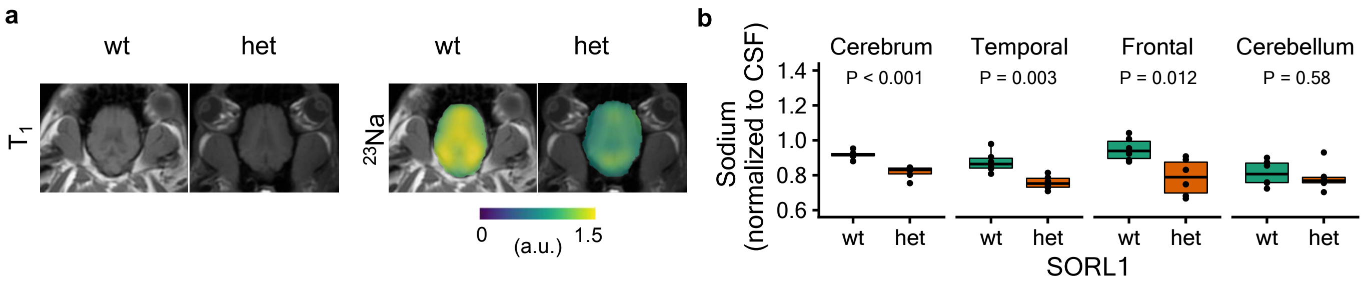

Figure 3: Sodium MRI of wild-type (*wt*) or *SORL1* haploinsufficient minipigs (*het*). The normalized sodium signal (**a**) was decreased in the brain of *SORL1*-deficient pigs (*het*), while no difference between *het* and *wt* animals was observed in the cerebellum (**b**). Extracerebral signal was cropped for display. T-tests were used for assessment of statistical significance.

For Review Only

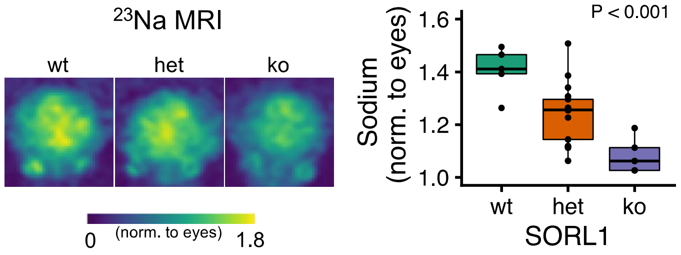

Figure 4: Sodium MRI images of wild-type (*wt*) mice and mice heterozygous (*het*) or homozygous (*ko*) for *SORL1* causing SORLA deficiency. The normalized sodium signal across the brain was decreased in a gene dose-dependent manner. Statistical significance was assessed using ANOVA.

For Review Only

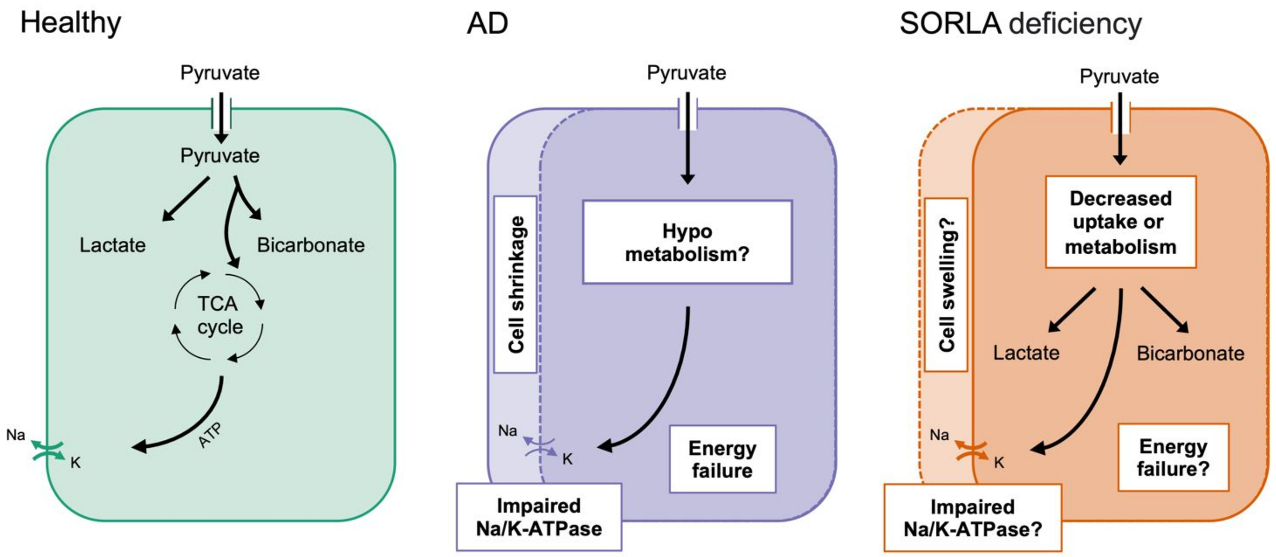

Figure 5: Schematic of the imaged metabolic changes in *SORL1*-deficient animals and potential underlying mechanisms in comparison to Alzheimer's disease (AD). The energy requirements of a healthy brain are fulfilled by glucose oxidation and glycolysis, and much of this energy is spend on maintaining the sodium gradient across the cell membrane. Hypometabolism is a typical feature of AD, hypothesized to cause failure in maintaining the sodium gradient and, in combination with cell death, leading to an increased ^{23}Na-MRI signal.[23,24] In the *SORL1*-deficient animals of this study, we observed a decrease in pyruvate-to-lactate and pyruvate-to-bicarbonate conversion coupled with a decrease in apparent ^{23}Na. The former could be due to decreased pyruvate uptake or metabolism, while the latter could be caused by cellular volume changes regardless of energy status.

For Review Only
