# Supplementary material for: Mice and minipigs with compromised expression of the Alzheimer’s disease gene SORL1 show cerebral metabolic disturbances on hyperpolarized [1-13C]pyruvate and sodium MRI
Source: Brain Commun. 2024 Mar 31;6(2):fcae114. doi: 10.1093/braincomms/fcae114 (PMC11034025; doi:10.1093/braincomms/fcae114)
Supplement: fcae114_Supplementary_Data [file Original Submission - Author's Response.pdf › Original Submission - Author's Response.pdf]

We would first like to thank the reviewers and editors for providing and facilitating a quick and helpful review. We are happy to learn that you find our paper important and sound. Please find our response to the reviewers and editorial comments below. They are given in a point-by-point fashion, and the revisions in the manuscript are highlighted in red.

Reviewer: 1

Comments to the Author
This study of sodium and hyperpolarized 13C MRI in an animal model of early AD is well designed and carried out. The model is an interesting one, with its ability to probe an aspect of biology and metabolism in AD which is not well understood.

The sodium results are surprising, suggesting a decrease in cerebral sodium whereas previous work has suggested an increase. The authors' arguments are persuasive that this may be due to the youth of the animals and asymptomatic phase of disease, and/or to cell swelling. It may be helpful for readers to follow this latter argument if some discussion is added of the typical intra- and extra-cellular concentrations of sodium in the brain (for example, perhaps they could be indicated in figure 5).

> *We have revised figure 5 as suggested, thank you for a helpful suggestion.*

More discussion might also be helpful of this statement on page 11: 'Decreased levels of pyruvate uptake would explain the equivalent decrease of pyruvate-to-lactate and pyruvate-to-bicarbonate exchange that we observed.' I think the rationale here is that pyruvate in the tissue would be lower but pyruvate in the bloodstream would still be detected at the same levels, so the metabolite ratios to pyruvate would be decreased regardless of the metabolic pathways involved. The authors should clarify.

> *The rationale is perfectly well-understood. We have revised the sentence to improve readability, thank you.*

There are a few small typos and places where the syntax could be improved, but nothing that obscures the meaning.

> *We have revised for typos and clarity throughout.*

Reviewer: 2

Comments to the Author
SORL1 mutations have been recognized as causal for familial and sporadic forms of Alzheimer Disease (AD). On the cellular level, loss of SORL1 causes endosomal abnormalities, whereas on the overall physiological level SORL1 has been suggested to regulate metabolic functions. However, at both levels, there is a lack of comprehensive

knowledge to fully explain Sorl1 function.
In this manuscript, Bogh and colleagues assess in vivo if decreased SORL1 levels cause metabolic dysfunction in the brain. To this end, they engage metabolic MRI. On one hand MRI with hyperpolarized [1-13C]pyruvate and on the other MRI of 23Na.

They use in their study two animal models, SORL1 knockout mice (wild type, hetero-, and homozygous) and Göttingen minipigs (wild type and heterozygous).
The minipig model was recently described for the first time, and so far, there appears to be only one heterozygous cohort and no homozygous KOs. Interestingly, the SORL1 heterozygous pigs did not display neurodegenerative changes in histology or structural analyzes, but show amyloid build-up in the cerebrospinal fluid and are thought to correspond to a preclinical stage of AD in humans.
The current manuscript describes that the lactate-to-pyruvate ratio, reflecting pyruvate uptake and glycolysis, was significantly decreased across the brain and frontal cortex in the SORL1-heterozygous minipigs. Although statistically not all significant, there was also a clear trend in the frontal and temporal cortex whereas there were no clear-cut changes in the cerebellum.
In the SORL1-heterozygous minipigs, they found a decrease in the total whole-brain sodium signal. This was also observed in the frontal and temporal cortex whereas there were no changes in the cerebellum.
The whole-brain sodium signal results were confirmed in the SORL1 mouse model with a graduated reduction from wt to heterozygous to homozygous animals.
The results of this study are interesting and relevant because they indicate metabolic changes caused by reduced SORL1 levels and support its metabolic function. The results are also highly significant because they link genetic alterations underlying AD to metabolic brain dysfunction at a likely preclinical stage of AD. These brain dysfunctions may also be detectable in patients. This translational aspect makes the current study very valuable.

Thank you very much for recognizing the significance and relevance of the study.

I am not a specialist in metabolic MRI, but overall the study is sound.
However, I have a number of concerns pertaining presentation and interpretation of data and the use of terminology and wording.
1. The pigs are not SORL1 deficient as stated several times e.g. in fig legend 3, they are heterozygous animals with strongly reduced SORL1 levels and calling them SORL1 deficient will lead to misinterpretation of the data by superficial readers.

This is a good point, thank you. In effort to improve readability, we have substituted 'deficient' throughout.

2. The authors should clearly emphasize why they could not use homozygous SORL1 minipigs.

This is addressed in revised 'Animals' section. The reason is simply that the heterozygous animals reflect SORL1 AD patients with SORL1 mutations well.

Also, to breed homozygous SORL1 knockout minipigs specifically for this study would require considerably more financial funding and experimental time than is the case for the rodents. We therefore chose to include only wildtype and the clinically relevant heterozygous genotypes for the pig model in our study.

3. Results section, it is sometimes not immediately clear which animal model is described. Please point this out directly at the beginning of each section.

The species is now explicitly stated in the beginning of each results section as well as in the sub-heading, thank you.

4. Results section, first paragraph, the authors conclude: "The lactate-to-bicarbonate ratio, reflecting the balance between glycolytic and oxidative metabolism, was similar between wt and het pigs." This statement contradicts the description of the results where changes were observed and to several statements in the discussion. Please revise.

We believe that this concern stems from our unclear description of the ratio presented in the cited statement. Accordingly, we have revised the statement to explicitly state that it describes the ratio between lactate and bicarbonate, or a combination of the metabolite-to-pyruvate ratios otherwise described earlier in the results section. Thank you for providing an opportunity to clarify.

Comments from the Scientific Editorial Team:

1. Please provide details of the **genders** of the animals in the study and whether the study was powered to examine sex effects.

   This is now described in the 'Animals' section.

2. Please ensure all abbreviations used in figures and figure legends are defined within each legend, so that they may be easily interpreted as a stand-alone figure.

   We have double-checked this and added the missing abbreviations.

3. Figure 1. B: Please label the heatmap scale and add a scale bar to this figure.

   We have made the requested additions, thank you.

4. Figure 2, 3. B, and 4. B: Please state what each individual data point represents in the figure legend.

   In all cases, a single point is a single animal, which is now mentioned in the legend.

5. Figure 3. A and 4. A: Please add a scale bar.

    These have been added, thank you.

6. Please add an **Abbreviated Summary** (up to 50 words) of your paper that captures the main purpose and conclusions of the work. The summary should state the main results written in the third person ('[1st author surname] et al. report that ...') and conclusion. This summary will be used in the contents list online and should not contain any abbreviations other than those on the accepted abbreviations list and accepted gene/protein names, making it accessible to the non-specialist. Please upload it as a Word document. Allowed abbreviations: AIDS; ANOVA; AMPA; ATP; A,T,C,G; CNS; CSF; CT; DNA; ECG; EEG; EMG; GABA; HIV; MRI; NMDA; PET; PCR; RNA.

    This has been added to the revised manuscript, thank you.

7. Please supply a **graphical abstract**. A graphical abstract is a visual representation of the central finding or methodology of the paper. It will be displayed in the table of contents. Authors should strive to make the graphical abstract informative, interesting, visually appealing, and straightforward (no caption needed, no abbreviations except gene names).

    We provide a graphical abstract in the revision.
