# Supplementary material for: Mice and minipigs with compromised expression of the Alzheimer’s disease gene SORL1 show cerebral metabolic disturbances on hyperpolarized [1-13C]pyruvate and sodium MRI
Source: Brain Commun. 2024 Mar 31;6(2):fcae114. doi: 10.1093/braincomms/fcae114 (PMC11034025; doi:10.1093/braincomms/fcae114)
Supplement: fcae114_Supplementary_Data [file Original Submission - Decision Letter.pdf › Original Submission - Decision Letter.pdf]

Original Submission - Decision Letter

11-Jan-2024

Dear Dr. Bøgh,

Thank you for your recent submission BRAINCOM-2023-555
entitled "SORLA-deficient mice and pigs show cerebral metabolic disturbances on 23Na and hyperpolarized [1-13C]pyruvate MRI" to Brain Communications.

Your manuscript has been reviewed, and while the reviewers had largely positive comments, they have some constructive critiques that I think you could address in a minor revision of the manuscript. Therefore, I invite you to respond to the reviewer(s)' comments and revise your manuscript. The reviewer(s)' comments are included at the bottom of this message.

In addition to the reviewers' comments, please also address our Scientific Editor's comments added below to comply with our requirements for presentation of scientific data at Brain Communications.

Please format your revised manuscript according to our Instructions to Authors which can be found at: https://academic.oup.com/braincomms/pages/General_Instructions
A summary of key points is attached to help with this. Please note that there can be substantial delays to publication if you don't resubmit in line with our requirements.

To revise your manuscript, click the link below or log into https://mc.manuscriptcentral.com/braincom and enter your Author Centre, where you will find your manuscript title listed under "Manuscripts with Decisions." Under "Actions," click on "Create a Revision." Your manuscript number will be appended to denote a revision.

*** PLEASE NOTE: This is a two-step process. After clicking on the link, you will be directed to a webpage to confirm. ***

https://mc.manuscriptcentral.com/braincom?URL_MASK=2fbcda8e58ab4b298a4fc2171e6bc64e

Please upload a revised manuscript with the changes you have made shown either in coloured text or using "track changes".

When submitting your revised manuscript, you will be asked to respond to the reviewer(s) comments. Please be as specific as possible in your response to all of the comments.

The editorial team at Brain Communications also aims to increase transparency in scientific publishing and to demonstrate the value that peer review brings to the scientific process. Please therefore consider whether you are willing to have reviewer comments on your paper and your response published as supplementary data with the paper, if it is accepted. This is entirely optional and will only be done if both the authors and reviewers agree. When submitting your revision you will have the opportunity to update the answer you entered previously.

IMPORTANT: Your original files are available to you when you upload your revised manuscript. Please delete any redundant files before completing the submission.

Because we are trying to facilitate timely publication of manuscripts submitted to Brain Communications, we would appreciate your revised manuscript as soon as possible. If it is not possible for you to resubmit within 3 months, the option to submit a revision will expire, and we would then consider your revised paper as a new submission. However, if you need a little more time, please get in touch and we can extend this deadline for you.

Once again, thank you for submitting your manuscript to Brain Communications and I look forward to receiving your revision.

Best wishes

Dr. Paul Skehel
Associate Editor, Brain Communications

Professor Tara Spires-Jones
Founding Editor, Brain Communications

Reviewer(s)' Comments to Author:

Reviewer: 1 (Mary McLean)

Comments to the Author
This study of sodium and hyperpolarized 13C MRI in an animal model of early AD is well designed and carried out. The model is an interesting one, with its ability to probe an aspect of biology and metabolism in AD which is not well understood.

The sodium results are surprising, suggesting a decrease in cerebral sodium whereas previous work has suggested an increase. The authors' arguments are persuasive that this may be due to the youth of the animals and asymptomatic phase of disease, and/or to cell swelling. It may be helpful for readers to follow this latter argument if some discussion is added of the typical intra- and extra-cellular concentrations of sodium in the brain (for example, perhaps they could be indicated in figure 5).

More discussion might also be helpful of this statement on page 11: 'Decreased levels of pyruvate uptake would explain the equivalent decrease of pyruvate-to-lactate and pyruvate-to-bicarbonate exchange that we observed.' I think the rationale here is that pyruvate in the tissue would be lower but pyruvate in the bloodstream would still be detected at the same levels, so the metabolite ratios to pyruvate would be decreased regardless of the metabolic pathways involved. The authors should clarify.

There are a few small typos and places where the syntax could be improved, but nothing that obscures the meaning.

Reviewer: 2

Comments to the Author
SORL1 mutations have been recognized as causal for familial and sporadic forms of Alzheimer Disease (AD). On the cellular level, loss of SORL1 causes endosomal abnormalities, whereas on the overall physiological level SORL1 has been suggested to regulate metabolic functions. However, at both levels, there is a lack of comprehensive knowledge to fully explain Sorl1 function.

In this manuscript, Bogh and colleagues assess in vivo if decreased SORL1 levels cause metabolic dysfunction in the brain. To this end, they engage metabolic MRI. On one hand MRI with hyperpolarized [1-13C]pyruvate and on the other MRI of 23Na.

They use in their study two animal models, SORL1 knockout mice (wild type, hetero-, and homozygous) and Göttingen minipigs (wild type and heterozygous).

The minipig model was recently described for the first time, and so far, there appears to be only one heterozygous cohort and no homozygous KOs. Interestingly, the SORL1 heterozygous pigs did not display neurodegenerative changes in histology or structural analyzes, but show amyloid build-up in the cerebrospinal fluid and are thought to correspond to a preclinical stage of AD in humans.

The current manuscript describes that the lactate-to-pyruvate ratio, reflecting pyruvate uptake and glycolysis, was significantly decreased across the brain and frontal cortex in the SORL1-heterozygous minipigs. Although statistically not all significant, there was also a clear trend in the frontal and temporal cortex whereas there were no clear-cut changes in the cerebellum.

In the SORL1-heterozygous minipigs, they found a decrease in the total whole-brain sodium signal. This was also observed in the frontal and temporal cortex whereas there were no changes in the cerebellum.
The whole-brain sodium signal results were confirmed in the SORL1 mouse model with a graduated reduction from wt to heterozygous to homozygous animals.

The results of this study are interesting and relevant because they indicate metabolic changes caused by reduced SORL1 levels and support its metabolic function. The results are also highly significant because they link genetic alterations underlying AD to metabolic brain dysfunction at a likely preclinical stage of AD. These brain dysfunctions may also be detectable in patients. This translational aspect makes the current study very valuable.

I am not a specialist in metabolic MRI, but overall the study is sound.
However, I have a number of concerns pertaining presentation and interpretation of data and the use of terminology and wording.
1. The pigs are not SORL1 deficient as stated several times e.g. in fig legend 3, they are heterozygous animals with strongly reduced SORL1 levels and calling them SORL1 deficient will lead to misinterpretation of the data by superficial readers.
2. The authors should clearly emphasize why they could not use homozygous SORL1 minipigs.
3. Results section, it is sometimes not immediately clear which animal model is described. Please point this out directly at the beginning of each section.
4. Results section, first paragraph, the authors conclude: “The lactate-to-bicarbonate ratio, reflecting the balance between glycolytic and oxidative metabolism, was similar between wt and het pigs.” This statement contradicts the description of the results where changes were observed and to several statements in the discussion. Please revise.

Comments from the Scientific Editorial Team:
Please provide details of the genders of the animals in the study and whether the study was powered to examine sex effects.
Please ensure all abbreviations used in figures and figure legends are defined within each legend, so that they may be easily interpreted as a stand-alone figure.
Figure 1. B: Please label the heatmap scale and add a scale bar to this figure.
Figure 2, 3. B, and 4. B: Please state what each individual data point represents in the figure legend.
Figure 3. A and 4. A: Please add a scale bar.
Please add an Abbreviated Summary (up to 50 words) of your paper that captures the main purpose and conclusions of the work. The summary should state the main results written in the third person (‘[1st author surname] et al. report that …’) and conclusion. This summary will be used in the contents list online and should not contain any abbreviations other than those on the accepted abbreviations list and accepted gene/protein names, making it accessible to the non-specialist. Please upload it as a Word document. Allowed abbreviations: AIDS; ANOVA; AMPA; ATP; A,T,C,G; CNS; CSF; CT; DNA; ECG; EEG; EMG; GABA; HIV; MRI; NMDA; PET; PCR; RNA.
Please supply a graphical abstract. A graphical abstract is a visual representation of the central finding or methodology of the paper. It will be displayed in the table of contents. Authors should strive to make the graphical abstract informative, interesting, visually appealing, and straightforward (no caption needed, no abbreviations except gene names). The technical requirements for the graphical abstracts are:
- Size: 120 x 120 mm (300-600dpi) or 3000 x 3000 pixels
- Font:Arial 12-16 pt
- File type: tiff or PDF
- Aspect ratio: 1:1 preferred
